# Validity and Reliability of the Malay Version of the Identification of Functional Ankle Instability (IdFAI-M) Questionnaire among Malaysian University Athletes

**DOI:** 10.5704/MOJ.2103.006

**Published:** 2021-03

**Authors:** MK Omar, S Abdul-Karim

**Affiliations:** Department of Sport Medicine, University of Malaya, Kuala Lumpur, Malaysia

**Keywords:** functional ankle instability, translation, validation, reliability, IdFAI

## Abstract

**Introduction:**

This study was designed to produce a validated and reliable Malay version of the Identification of Functional Ankle Instability (IdFAI-M) questionnaire.

**Materials and method:**

The cross-cultural adaptation was conducted based on standard guidelines to produce the Malay version of the Identification of Functional Ankle Instability (IdFAI-M) questionnaire. The reliability and validity testing were then performed among one hundred and twenty-three physically active University of Malaya students. Among them, twenty-two students also participated in the second return of the questionnaire over a two-week interval, which was then evaluated for test-retest reliability testing.

**Results:**

The content validity for item-level (I-CVI) and Kappa values for all items were more than 0.7, respectively and the all scales-level (S-CVI) values were 0.983 (consistency), 0.967 (representativeness), 1.00 (relevance) and 0.983 (clarity). The questionnaire also demonstrated excellent reliability with an intraclass correlation coefficient (ICC2.1) above 0.850 for all items. It was observed that outer loading of most items were more than the minimum acceptable value (0.7). Fornell-Larcker criterion demonstrate all values for each reflective construct was larger than the correlations with other constructs, indicating discriminant. The cross-loading values of each item has shown a weak correlation with all other constructs, except for the one to which it was theoretically associated.

**Conclusions:**

The Malay version of the IdFAI (IdFAI-M) is a reliable and valid instrument that can be readily utilised to subjectively assess ankle instability.

## Introduction

Long term sequelae of ankle injuries are varied. Approximately 73% of all athletes had recurrent ankle sprain, and 59% of these athletes had significant disability and residual symptoms resulting in athletic performance impairment^1^. These residual symptoms include pain, weakness, recurrence sprain and instability^2,3^. This occurrence of repeated lateral ankle sprains is known as chronic ankle instability (CAI) and characterised by a multifactorial condition involving mechanical and/or functional instability^4,5^. Functional ankle instability (FAI) is defined as a joint motion that did not necessarily exceed normal physiological limits but was beyond voluntary control^6^. However, the definition of FAI has always been difficult due to the subjective feeling of the sufferers. The earliest author to come up with this definition was Freeman in 1965, which he described as “tendency for the foot to ‘give way’ after an initial ankle sprain”^7^.

Few self-reporting measures have been used to assess the FAI found in the literature^8-14^. In a systematic review by Donahue *et al*, only the Cumberland Ankle Instability (CAIT) and Ankle Instability Instrument (AII) questionnaires were the only statistically significant predictors of FAI status^15^. Hence, Simon *et al* (2012) has developed a new set of self-measure questionnaire, named the Identification of Functional Ankle Instability (IdFAI). The IdFAI has shown to have a distinct discriminative validity and an accuracy of 89.6% with a calculated sensitivity of 0.83 and specificity of 0.94^16,17^. The specific definition of ‘giving way’; a temporary uncontrollable sensation of instability or rolling over of one’s ankle’, is also mentioned at the top of the questionnaire, which allow patients to have better understanding regarding the questions.

**Fig. 1:**
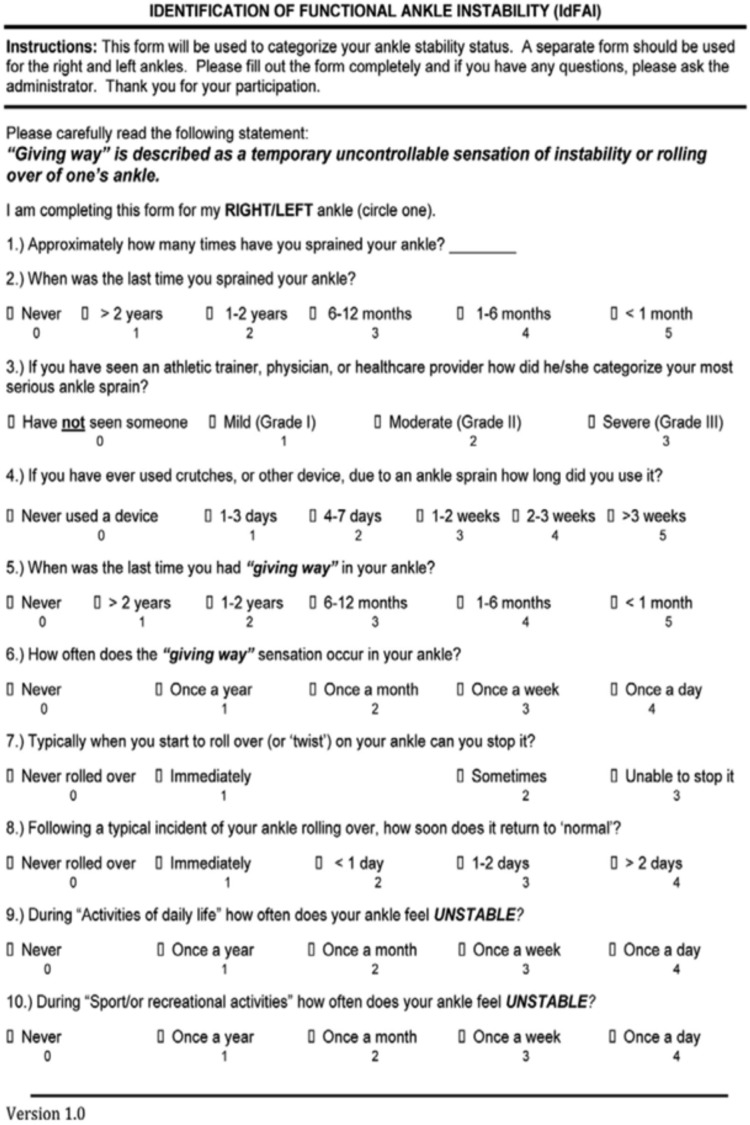
The Identification of Functional Ankle Instability.

The instrument, developed in English, was widely accepted and used in several investigations. However, multinational and multicultural research is on the rise, hence cross-cultural questionnaire adaptation is required. The IdFAI (Fig. 1) has been translated and culturally adapted into different languages including Korean, Mandarin and Portuguese^18-20^. Thus, a systematic process of cultural adaptation and validation is required to provide a valid IdFAI questionnaire for the Malay-speaking population. It is important for the questionnaire to be comprehensible and relevant in the new setting, and this process is called cross-cultural adaptation^21^. Hence, the aim of this study is to translate and cross-culturally adapt the Malay version of IdFAI (IdFAI-M) questionnaire and to assess its validity and reliability of the new version.

## Material and Method

This study started with the development of the study protocol, which was conducted by the research team and proceeded with the application of ethics and approval. A linguistic translation and cultural adaptation of the IdFAI from English to Malay was then performed and a prospective instrument validation and reliability study was then carried out.

For translation procedure, this study was approved by the University Malaya Medical Centre (UMMC) ethics committee and complied with the Declaration of Helsinki. Approval to translate the original version of the questionnaire had been obtained from the author. The English version of the IdFAI questionnaire was adapted for use by the general population of Malaysia in accordance with the recommendations for cross-cultural adaptation of the six-stage self-reporting measures^22-26^.

Stage 1 is forward translation by which three bilingual translators who were well versed in the Malay and English languages, generated separate translations. They were a sports physician, a sports medicine medical officer and a secondary school teacher with a major in physical education (PE). The sports physician and the medical officer were aware of the concepts being examined in the questionnaires to be translated. Their translations were intended to provide equivalence from a more clinical and psychological point of view. On the other hand, the schoolteacher was neither aware of the concepts that were quantified in such a way that he would be less influenced by the academic goal and offered a translation that reflects the language used by athletes and the general public. Each of them translated the questionnaire individually and three translated Malay versions were produced (M1, M2 and M3). In Stage 2 (synthesis), the researcher and team produced a common translation transcript (M4) of the three translated questionnaires (M4). Stage 3 is back translation, which involved three different translators (a sports physician, a sports medical officer and an English schoolteacher) with English as their primary language, translated the M4 back to the original language (English). The translators were completely blinded to the original version and the three back-translations (E1, E2 and E3) were produced. A committee consisting of experts in musculoskeletal conditions and applied language reviewed all translations and reached agreement on any differences. The material at the disposal of the committee included the original questionnaire, and each translation (M1, M2, M3, M4, E1, E2 and E3) together with corresponding written reports, if available. Semantic, idiomatic, experiential, conceptual and functional equivalences were reached for all differences in the pre-final version of the translated version of Malay questionnaire^23,26^. Stage 5 was the pre-test of the process of adaptation in which 30 random students were tested^24^. Thirty university athletes completed the questionnaire and verbally informed the author of what he or she thought was meant by each item of the questionnaire and the response chosen. The instrument's content validity was also assessed using the panel of experts' perspectives during this stage. Taking these into account, a final Malay version of IdFAI (IdFAI-M) was developed. The final stage was submission of documentation to the developers or coordinating committee for appraisal of the adaptation process. The IdFAI-M questionnaire developers reviewed the final version of the IdFAI-M and all reports covering Stage 1 through 5 and analysed the process of adaptation. At this point, the IdFAI-M questionnaire was finally ready to be tested for its validity and reliability among participants in University of Malaya (UM).

For validity and reliability of the questionnaire, this study was conducted in UM. Students with the following criteria were recruited: aged above 18 years old and recreationally active, i.e. performing physical or sports activities on a weekly basis for at least 90 minutes. The exclusion criteria were participants with a history of head, spine and/or lower extremity injury within the past six months, a history of lower extremity surgery and any neurological, neuropsychiatric, vestibular, or other connective tissue disorders^18^. The sample size was calculated from a statistical aspect based on the guidelines for the respondent-to-item ratio which ranged from 5:1 (i.e. 50 respondents for a 10-item questionnaire), 10:1, to 15:1 or 30:1^27^. Therefore, the required sample size was 50 or 100, as per respondent-to-item ratios of 5:1 and 10:1, respectively in which the IdFAI-M questionnaire consisted of 10 items. However, we gathered data from one hundred and twenty-three (123) students, thereby improving the ratio to 12:1. The sample size for test-retest for estimating the value of intraclass correlation coefficient is 28 with the alpha and minimum required power is fixed at 0.05 and higher than 0.80, respectively and an additional 20% of drop-out rate is also included. This calculation was derived from Power Analysis and Sample Size (PASS) software^28^. The sampling method used for both samples are convenience sampling.

**Fig. 2:**
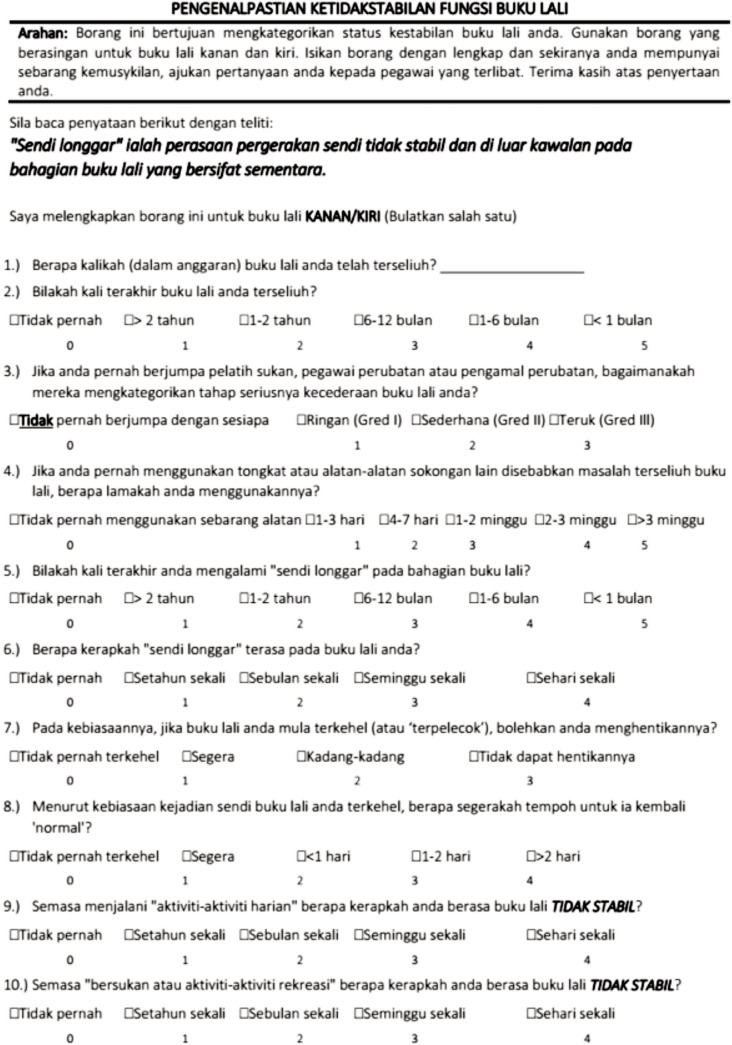
The Malay Version of the Identification of Functional Ankle Instabily (IdFAI-M).

Information about participant characteristics (e.g. age, race, gender, height, and weight) and sports involvement profile (e.g. type of sports, level of participation and years of experience) were recorded in a form. The final Malay version of the IdFAI questionnaire (Fig. 2) was distributed to the participants. This questionnaire was tested in both ankles. The one with the highest functional instability score was chosen for analysis. IdFAI scores may vary from 0 to 37, suggesting that individuals with a score of less than 10, do not have FAI and those with a score of eleven or above do have^16^. After a two weeks interval, twenty-two participants randomly selected and completed a different set of IdFAI-M again.

For validity test, content and construct validity were performed. For content validity, the process involved the panel of experts to rate the clarity, representativeness, consistency and relevance of each item of the translated version using a four-point ordinal (1 not relevant, 2 somewhat relevant, 3 quite relevant, 4 highly relevant) scale. The content validity index (CVI) of each item was calculated, based on their ratings^29^.

For construct validity, which an instrument measures the trait or theoretical construct that it is intended to measure^30^. We tested on convergent and discriminant validity. Convergent validity is the evaluation to measure the level of correlation between multiple indicators of the same construct in agreement. The factor loading of the indicator, composite reliability (CR) and average variance extracted (AVE) must be considered in order to establish convergent validity. Discriminant validity refers to the extent that the construct is empirically different from each other. It also measures the degree to which the overlapping structure differs. A cross-loading indicator and Fornell and Larcker criterion were used to test the discriminant validity^31^.

Test-retest reliability is an extension of how similar results can be achieved with different administrations and was assessed with an intraclass correlation coefficient (ICC2.1). The IdFAI-M was administered twice with 22 participants had answered the IdFAI-M after two weeks from the first return^30,32,33^. An ICC2.1 equal to or greater than 0.70 was considered reasonable for test-retest reliability^34^. Composite reliability has also been calculated to determine the internal consistency in which it measured the reliability on the basis of the interrelationship of variable items observed. In exploratory research, the values of composite reliability between 0.60 and 0.70 are acceptable, while at a more advanced stage the values must be higher than 0.70. However, the value that is more than 0.90 is definitely undesirable^31^.

Cohen's kappa coefficient (κ) is a statistic that is used to measure inter-rater reliability for qualitative (categorical) items during the content validity analysis. It is the degree of concordance between two or more raters (experts) for the instrument scores. Two qualified raters are expected to have the same score in the same instrument, so Kappa will measure this inter-observer reliability^35^. The Kappa result is interpreted as follows: values between 0 and 0.20 imply no agreement and between 0.21 and 0.39 as a minimum, between 0.40 and 0.59 as weak, between 0.60 and 0.79 as moderate, between 0.80 and 0.90 as strong and above 90 as nearly perfect agreement^36^.

All the analyses were conducted using the SPSS [Version 24.0; IBM Corp, Armonk, NY, USA] and smart-PLS version 3.2.8.

## Results

One hundred and twenty-three university students aged 18-year-old and above were involved in this study. They participated in various types of sports with differing levels of participation. The median age of the respondents was 21 (IQR 4) years, of which 55.3% were male. The demographic representation of the respondents is listed in Table I. Of the 123 respondents, only 22 students completed the questionnaire after two weeks of the first administration (18%).

**Table I:** Descriptive profile of the study participants (n=123)

Age (year), median (IQR)	21 (4)
Variables	Frequency, n (%)
Gender	
Male	68 (55.3)
Female	55 (44.7)
Race	
Malay	81 (65.9)
Chinese	31 (25.2)
Indian	6 (4.9)
Others	5 (4.1)
Height (metre), mean (SD)	1.66 (±0.92)
Weight (kilogram), mean (SD)	62.49 (±15.024)
Variables	Frequency, n (%)
BMI, mean (SD)	22.51 (±4.057)
Primary language	
Malay	84 (68.3)
Non-Malay	39 (31.7)
Faculty	
Medicine	55 (44.7)
Others	68 (55.3)
Type of sports	
Contact	29 (23.6)
Limited contact	24 (19.5)
Non-contact	70 (56.9)
Level of highest participation in sports activity	
College	35 (28.5)
University	34 (27.6)
State	14 (11.4)
International	11 (8.9)
Not related	29 (23.6)
Side of ankle being tested	
Right	92 (74.8)
Left	25.2)

The content validity for item-level (I-CVI) and Kappa values for all the aforementioned items were more than 0.79 and 0.74, respectively and the content validity for all scales-level (S-CVI) values are 0.983 (consistency), 0.967 (representativeness), 1.00 (relevance) and 0.983 (clarity) Table II. Table III shows the result of construct validity test which the outer loading of all item numbers except four and seven were more than minimum acceptable value (0.7). Loadings above 0.7 implied that the indicators have much in common, which was desirable for reflective measurement models^31^. The average variance extracted (AVE) indicates convergent validity for a construct, and the value for each reflective construct were 0.682, 0.488 and 0.672 Table III. A threshold of 0.5 is acceptable, indicating that the construct explains at least half of the variance of the indicators^37^.

**Table II:** Content validity for item-level (I-CVI), scale-level (S-CVI) and Kappa values for each item

Item	Consistency		Representativeness		Relevance		Clarity		Result
	CVI	Kappa	CVI	Kappa	CVI	Kappa	CVI	Kappa	
1	1.000	1.000	1.000	1.000	1.000	1.000	1.000	1.000	Validated
2	1.000	1.000	1.000	1.000	1.000	1.000	1.000	1.000	Validated
3	1.000	1.000	1.000	1.000	1.000	1.000	0.833	0.816	Validated
4	1.000	1.000	1.000	1.000	1.000	1.000	1.000	1.000	Validated
5	1.000	1.000	0.833	0.816	1.000	1.000	1.000	1.000	Validated
6	1.000	1.000	1.000	1.000	1.000	1.000	1.000	1.000	Validated
7	0.833	0.816	1.000	1.000	1.000	1.000	1.000	1.000	Validated
8	1.000	1.000	1.000	1.000	1.000	1.000	1.000	1.000	Validated
9	1.000	1.000	0.833	0.816	1.000	1.000	1.000	1.000	Validated
10	1.000	1.000	1.000	1.000	1.000	1.000	1.000	1.000	Validated
S-CVI	0.983		0.967		1.000		0.983		

**Table III:** Composite reliability, the average variance extracted (AVE) and outer loading between constructs and items

Construct/Factor	Item	Outer Loading	Composite Reliability	AVE
History of ankle instability	Q10	0.855	0.894	0.682
	Q5	0.872		
	Q6	0.894		
	Q7	0.661		
Initial ankle sprain	Q2	0.789	0.738	0.488
	Q3	0.719		
	Q4	0.570		
Instability during activities	Q8	0.778	0.804	0.673
	Q9	0.861		

Discriminant validity was checked based on Fornell-Larcker criterion and cross loading value. Table IV displays the square root of the AVE on the diagonal in parentheses. All values for each reflective construct were larger than the correlations with other constructs, indicating discriminant. The cross-loading values of each item Table V has shown a weak correlation with all other construct, except for the one to which it was theoretically associated except for the item number 7, which had a stronger correlation with factor 3 (0.667) when comparing to its own construct (0.661).

Table VI presents the test-retest reliability result. There were no major intra-individual disagreements in the test-retest answers upon the first and the second return. The questionnaire demonstrated excellent reliability with an intraclass correlation coefficient (ICC2.1) of 0.973, 0.836, 0.949, 0.857, 0.949, 0.938, 0.905, 0.804, 0.784 and 0.978 with value of p<0.001, for items 1 till 10, respectively. This indicated that the IdFAI-M was highly stable across testing occasions. Conversely, the composite reliability Table III of all the reflective constructs were above 0.7. This demonstrated high levels of internal consistency reliability for all three reflective constructs.

**Table IV:** The square root of the average variance extracted (AVE)

	History of ankle instability	Initial ankle sprain	Instability during activities
History of ankle instability	0.826		
Initial ankle sprain	0.609	0.699	
Instability during activities	0.81	0.596	0.82

**Table V:** The cross-loading value between each item and the constructs

Item	History of ankle instability	Initial ankle sprain	Instability during activities
Q10	0.855	0.513	0.788
Q5	0.872	0.542	0.607
Q6	0.894	0.545	0.606
Q7	0.661	0.398	0.677
Q2	0.612	0.789	0.616
Q3	0.267	0.719	0.279
Q4	0.284	0.57	0.225
Q8	0.546	0.47	0.778
Q9	0.765	0.508	0.861

**Table VI:** Intraclass coefficient correlation (ICC2.1) of the Malay version of the Identification of Functional Ankle Instability (IdFAI-M)

Item	ICC2.1
Q1	0.973
Q2	0.836
Q3	0.949
Q4	0.857
Q5	0.949
Q6	0.938
Q7	0.905
Q8	0.804
Q9	0.784
Q10	0.978
Factor 1	0.969
Factor 2	0.945
Factor 3	0.889
Total score	0.974

## Discussion

Many suggested cross-cultural adaptation guidelines and recommendations have been found in the literature. In this study, the standard six-stage translation was mainly adapted from Beaton with some modifications^24^. First, the number of translators and the background of the translators used. There were three different translators in each forward and backward translation stages (Stage I and III), whilst only two translators is suggested in the reference^38^. The decision to include the third translator, as seen in this study, was believed to help reduce the discrepancy between the previous two translators. During the backward translation stage, it was suggested that both translators should neither be aware nor be informed of the concepts explored and should preferably be without medical background. However, in this study there was only one translator with no medical background while the other translators were sports physicians, but they were both unaware of the concepts being explored.

Despite the fact that other studies have used an array of comparisons to evaluate the validity of these self-report questionnaires, this study did not utilise any other local version of self-reporting tools to assess the correlation with other instrument as there is no validated Malay version of related questionnaire found in literature. Mohamadi et al employed four other different tools which are already translated to Persian language when performing the validity testing. These include the Persian version of CAIT, FAOS and FAAM, as well as the Fear Avoidance Belief Questionnaire (FABQ) and the Tampa Scale of Kinesophobia (TSK)^39^. In other different studies, a validated Brazilian Portuguese, Korean and Chinese versions of CAIT have also been used in developing the validity^18-20^. There were other studies which had performed the validity testing among bilingual population using the original IdFAI for comparison^19,40^. All of these studies have shown good validity and reliability. In addition, during the development of the original IdFAI, Donahue has assessed a correlation between original IdFAI and the LEFS when evaluating the validity of the questionnaires^41^. Hence the construct validity of this study was determined from its convergent validity and discriminant validity^31^.

Convergent validity refers to the model’s ability to explain the indicator’s variance. The average variance extracted (AVE) and outer loading provide evidence for convergent validity^42^. It is worth noting that outer loading of all indicators of reflective constructs were more than the minimum desirable value of 0.731. The outer values for the question number 7 and number 4 were 0.661 and 0.57, respectively, they were still above 0.4 which is acceptable to not be removed from the questionnaire^43^. Furthermore, both questions are relevant to demonstrate severity of the ankle sprain that may lead to chronic instability. The AVE values for all reflective constructs were above 0.5, except for the factor 2 value, which was 0.488. In view of the difference being too unsubstantial (0.012), it can be ignored^44^. In different studies, the factor 2 had also showed a weak to moderate correlation between the history of initial ankle sprain factor and the LEFS questionnaire^20,41^. Therefore, the fourth and seventh item do not have to be necessarily deleted.

Cross-loading items represent prime candidates for removal from subsequent analysis with the goal of improving model fit. If two or more factors had almost comparable factor loading, this signified that the item was not specific and should be eliminated. This can be seen in question number 7 which has a stronger correlation with a factor 3 (0.677) when compared to its own construct (0.661). This could be due to the fact that item 7 (Factor 1) addresses how the respondent's ability to stop the 'rolling-over' of the ankle was also demanded in item 8 and item 9 (Factor 3). However, we decided not to remove the item from the questionnaire due to judgemental reason as elimination of that item will affect the questionnaire’s content validity and coherence^45^. Cambra-Fierro decided to maintain an item in his study, despite of a relatively low item–total correlation, and it was regarded to be theoretically relevant^46^. Overall, discriminant validity can be accepted for this measurement model and supports the discriminant validity between the constructs.

Based on the sample size calculation in estimating intraclass correlation coefficient, it should be at least 28 respondents to complete the questionnaire after two weeks interval. However, there were only 22 respondents returned the questionnaire during the second assessment. Some of the respondents were therefore not receptive during the follow-up, and few of the questionnaires returned only fourteen days after the first completion, which could affect the reliability of the test. Nevertheless, the statistical analysis had shown excellent test-retest reliability of the IdFAI-M between the first and the second administration, for each item, factors and the total score, with the value of intraclass coefficient correlation (ICC2.1) of more than 0.75. The ICC2.1 value of total score of IdFAI-M was 0.97, which was comparable to the ICC2.1 value of the original IdFAI, ICC2.1 0.92. The ICC2.1 value for factor 1, 2 and 3 were 0.97, 0.95 and 0.89, respectively, whereas the original IdFAI’s factors were 0.81, 0.94 and 0.83, respectively^41^. The ICC2.1 value for Chinese version of IdFAI was 0.97, which was equally reliable with this study^19^. Whilst all the previous related studies used Cronbach’s coefficient alpha to measure the internal consistency, this study utilised a comparison of composite reliability for each factor^18-20,40^. Even though Cronbach's coefficient alpha is the most widely used estimator of the reliability of tests and scales, it has been criticised as being a lower bound estimate of internal consistency rather than a true estimate. This is due to the assumptions of Cronbach’s alpha that the scale is unidimensional, adheres to tau equivalence and its items are on a continuous scale and normally distributed^47^. It can underestimate its true reliability by as much as 20%^48,49^. Conversely, this is not implied by composite reliability but takes into account the various loading factors of the items. Hence, composite reliability is a more suitable measure of internal consistency reliability. The composite reliability of three factors are above the 0.7 threshold value, thus demonstrating high levels of internal consistency reliability for all three reflective constructs^50^.

There were a few limitations observed in this study. The first was pertaining to the homogenous study population amongst university students which could limit its generalisability. Secondly, as approximately 45% of the respondents were from Faculty of Medicine, they may be naturally familiar with the general content of the study. Apart from that, the respondents were among the groups that were physically active. Due to the different level of physical activity and/or knowledge, this may have produced a disparity in the perception of responding to the questionnaire. Hence, a variety of background and with different groups of participants are necessary in future studies.

## Conclusion

The cross-culturally adapted IdFAI-M is a highly reliable and valid self-report questionnaire that can be used to assess ankle instability. It can therefore be applied in the future in the Malaysian context to evaluate, prevent, and rehabilitate patients in research-oriented studies as well as clinical practice.
